# Laser Indirect Ophthalmoscopy-Guided Transpupillary Thermotherapy Versus I-125 Plaque Brachytherapy for Choroidal Hemangioma

**DOI:** 10.3390/cancers17183087

**Published:** 2025-09-22

**Authors:** Rima Torosyan, Imad Jaradat, Reem AlJabari, Mona Mohammad, Ibrahim AlNawaiseh, Yacoub A. Yousef

**Affiliations:** 1 Department of Surgery (Ophthalmology), King Hussein Cancer Centre (KHCC), Amman 11941, Jordan; torosyanrima@gmail.com (R.T.); ra.11229@khcc.jo (R.A.); mt2972@yahoo.com (M.M.); ia.00146@khcc.jo (I.A.); 2 Department of Radiation Oncology (Ophthalmology), King Hussein Cancer Centre (KHCC), Amman 11941, Jordan; ijaradat@khcc.jo

**Keywords:** choroid, hemangioma, radioactive plaque brachytherapy, transpupillary thermotherapy

## Abstract

**Background:** Choroidal hemangioma, a rare benign vascular tumor, can cause visual loss due to subretinal fluid. Photodynamic therapy (PDT) with verteporfin has been the standard treatment, with plaque brachytherapy reserved for PDT failure. Verteporfin is unavailable in many regions in the Middle East, including Jordan, leaving plaque as the main alternative; however, plaque often leads to poor visual outcomes despite tumor control. To improve visual outcomes, we introduced transpupillary thermotherapy (TTT) via laser indirect ophthalmoscopy (LIO) as a practical, widely available, vision-preserving treatment. **Methods:** We retrospectively reviewed 13 patients with choroidal hemangioma treated at King Hussein Cancer Center. Patients received either plaque brachytherapy or LIO-guided TTT. Clinical data included visual acuity at baseline, tumor thickness reduction, subretinal fluid status, and visual outcome. **Results:** All patients had unilateral circumscribed choroidal hemangioma, and 10 (77%) were males. At diagnosis, the visual acuity was ≤0.5 in all patients (100%) and <0.1 in six (46%) patients. Seven patients (54%) received LIO-guided TTT and six (46%) underwent I-125 plaque brachytherapy. Tumor thickness was 3.0–5.0 mm in 12 (92%) cases; the median thickness in the I-125 plaque brachytherapy group was 4.5 mm (range, 4.5–5.0 mm), whereas in the LIO-guided TTT group it was 3.8 mm (range, 2.9–5.0 mm). At a median follow-up of 20 months (mean 24, range 12–48 months), five out of seven patients (71%) treated with TTT showed significant visual improvement, while the remaining two (29%) had stable vision; none experienced deterioration. In contrast, none of the six plaque-treated patients (0%) demonstrated any improvement in visual acuity; four remained stable and two worsened. This difference was statistically significant (*p* = 0.021). Tumor thickness was reduced in both groups, with a median reduction of −56% in the plaque group and −36% in the TTT group. All patients achieved complete resolution of subretinal fluid. **Conclusions:** LIO-guided TTT is an effective vision-preserving treatment for choroidal hemangioma. While both modalities-controlled tumor growth, only TTT resulted in significant visual improvement. This study demonstrates that LIO-guided TTT can replace plaque brachytherapy in regions where verteporfin (PDT) is unavailable, offering an accessible, practical, and superior alternative for preserving vision in patients with choroidal hemangioma.

## Simple Summary

This study evaluates the efficacy of laser indirect ophthalmoscopy-guided transpupillary thermotherapy (LIO-guided TTT) compared with I-125 plaque brachytherapy for the treatment of circumscribed choroidal hemangioma in a setting where photodynamic therapy (PDT) is unavailable. Thirteen patients were retrospectively reviewed, with seven receiving LIO-guided TTT and six treated with plaque brachytherapy. Both treatments achieved tumor control and resolution of subretinal fluid; however, LIO-guided TTT demonstrated superior visual outcomes, with 71% of patients showing significant improvement compared to none in the plaque group. These findings suggest that LIO- guided TTT is a safe, accessible, and vision-preserving alternative to plaque therapy in regions lacking PDT.

## 1. Introduction

Circumscribed choroidal hemangioma (CCH) is a rare benign vascular tumor that accounts for less than 1% of all choroidal tumors diagnosed clinically [1]. The incidence of uveal melanoma (the most common intraocular malignant tumor in adults) in Jordan is 1.39 new cases per year per one million population [2], but the exact incidence of choroidal hemangioma in the general population is not well-defined due to its more asymptomatic nature and incidental detection during routine ophthalmic examinations [3]. CCH most commonly presents sporadically in adulthood, predominantly affecting individuals in their third to fifth decades of life [1,3]. In a large case series including 458 patients, the mean age at diagnosis was approximately 43 years, with no significant gender predilection noted, ref. [1] and with no predilection for any specific ethnicity [3,4]. Unlike the diffuse form associated with Sturge–Weber syndrome, which often presents congenitally, CCH typically arises sporadically without systemic associations [4,5].

CCH usually remains asymptomatic, typically presenting in adulthood with visual symptoms resulting from exudative retinal detachment, cystoid macular edema, or subretinal fluid accumulation [3,6]. Though histologically benign, its location in the macular region and associated complications can lead to irreversible vision loss [6,7,8,9]. Historically, treatment options for symptomatic CCH have included photodynamic therapy (PDT), radioactive plaque brachytherapy (which is commonly used for choroid melanoma), and transpupillary thermotherapy (TTT) [6,10,11,12,13,14,15,16]. Among these, PDT is recognized for its ability to selectively occlude tumor vasculature with minimal collateral retinal damage, achieving tumor regression in up to 80–90% of cases and visual stabilization or improvement in approximately 70–85% of patients [6,7,11,17,18]. However, PDT may require multiple sessions with variable visual outcome [6,10], and it is not universally accessible because verteporfin has been unavailable on the market in many countries in the Middle East, including Jordan, for over 15 years, rendering PDT an inaccessible treatment option. Plaque brachytherapy is an effective treatment with reported success rates of tumor regression near 90% and visual preservation in about 60–70% of treated eyes with extramacular tumors [10,19]; however, it is an invasive and expensive treatment with a potential for radiation-related complications [10,15,19,20].

On the other hand, TTT is a less invasive alternative that induces hyperthermia within the tumor tissue. Studies have demonstrated visual improvement in 44% of eyes treated with TTT, tumor regression in 11–20%, and resolution of exudative retinal detachment in over 90% of cases [7,21,22,23,24,25]. Laser Indirect Ophthalmoscopy (LIO)-guided TTT, which is commonly used for management of retinoblastoma [26], is widely available and could offer a cost-effective, less invasive, and vision-preserving alternative to conventional modalities, particularly in resource-limited settings. This study presents LIO-guided TTT as a practical therapeutic approach for choroidal hemangiomas, assessing its clinical efficacy and visual outcomes. The aim of this study is to evaluate the efficacy of Laser Indirect Ophthalmoscopy (LIO)-guided TTT in management for choroid hemangioma in comparison to I-125 plaque brachytherapy.

## 2. Methods

This is a retrospective case series approved by the Institutional Review Board at King Hussein Cancer Center (25KHCC222). It involves 13 eyes of 13 consecutive patients diagnosed with choroidal hemangioma between January 2016 and December 2024 at the King Hussein Cancer Center. Data was acquired from patients’ medical charts, fundus photos, and radiology reports. Diagnosis was based on characteristic clinical features supported by multimodal imaging, including ultrasonography, fundus photography, and Optical Coherent Tomography (OCT).

Outcome measures included age at diagnosis, gender, survival, and laterality, visual acuity at presentation and at the last follow-up, tumor features, management, management outcome, and survival. Tumors were evaluated based on pre-treatment tumor thickness, presence of subretinal fluid, the tumor’s distance from the optic disc and fovea, post-treatment tumor thickness, change in tumor thickness, sub-retinal fluid reduction, and complications during the treatment and visual outcome.

Patients treated with brachytherapy were assessed for additional parameters, including plaque size and radiation dose, and patients treated by laser indirect ophthalmoscopy-guided transpupillary thermotherapy (LIO-guided TTT) were assessed for TTT power and duration.

None of the patients in either group received anti-VEGF therapy for the management of subretinal fluid.

### 2.1. Inclusion and Exclusion Criteria

The eligibility criteria for inclusion were patients with a clinical diagnosis of small size (thickness less than or equal to 5 mm) circumscribed choroidal hemangioma (CCH) that were treated either with laser indirect ophthalmoscopy-guided transpupillary thermotherapy (LIO-guided TTT) or with I-125 radioactive plaque brachytherapy and were followed for at least 1 year after treatment.

Exclusion criteria included eyes with diffuse choroidal hemangiomas, eyes with Sturge–Weber Syndrome, choroidal hemangiomas with thickness more than 5 mm, eyes treated with modalities other than LIO-guided TTT or plaque brachytherapy, cases with incomplete follow-up data, and cases in which observation was chosen as the management plan.

### 2.2. Statistical Analysis

We used descriptive statistics for categorical (frequencies; percentages) and continuous (mean; median) variables. Fisher’s exact test was used to assess the association between treatment modality (LIO-guided TTT vs. plaque brachytherapy) and clinical outcomes, including visual acuity improvement, tumor thickness reduction, and sub-retinal fluid resolution. A *p*-value of <0.05 was considered statistically significant.

## 3. Results

Twenty-one patients were diagnosed with choroid hemangioma, eight were excluded from this study (four were asymptomatic and did not receive any treatment, two had diffuse choroid hemangioma with Sturge–Weber disease, one had a large choroid melanoma with 6.5 mm thickness, and one lost follow-up without getting treatment). Our final analysis included 13 patients with symptomatic circumscribed choroidal hemangioma.

### 3.1. Demographics and Tumor Features

The median age at diagnosis was 42 years (range: 4–62 years). The tumor was in the right eye in five (38%) patients and in the left eye in eight (62%) patients and no one had bilateral choroidal hemangioma. Ten (77%) were males and three (23%) were females, yielding a male-to-female ratio of 3.3:1. From these 13 patients, 6 (46%) were treated with plaque brachytherapy and seven (54%) underwent trans-pupillary thermotherapy (TTT) guided by laser indirect ophthalmoscopy (LIO) (Table 1).

At diagnosis, visual acuity ranged from light perception to 0.5 decimal. Overall, 69% of patients had tumors located in the macular region, while 31% had extra-macular tumors. In the LIO-guided TTT group, the mean tumor thickness was 3.8 mm, while in the radioactive plaque brachytherapy group it was 4.7 mm. The median tumor thickness in the I-125 plaque brachytherapy group was 4.5 mm (range, 4.5–5.0 mm), whereas in the LIO-guided TTT group it was 3.8 mm (range, 2.9–5.0 mm). The median tumor distance from the fovea was 1.0 mm (range, 0–1.8 mm) in the I-125 plaque brachytherapy group and 0 mm (range, 0–3.6 mm) in the TTT group. The median tumor distance from the optic nerve head was 1.0 mm (range, 0.5–4.1 mm) in the plaque group and 0 mm (range, 0–2.36 mm) in the TTT group (Table 1). The area of subretinal fluid, however, was not quantitatively measured in either group.

### 3.2. Treatment Parameters

**Figure 1:**
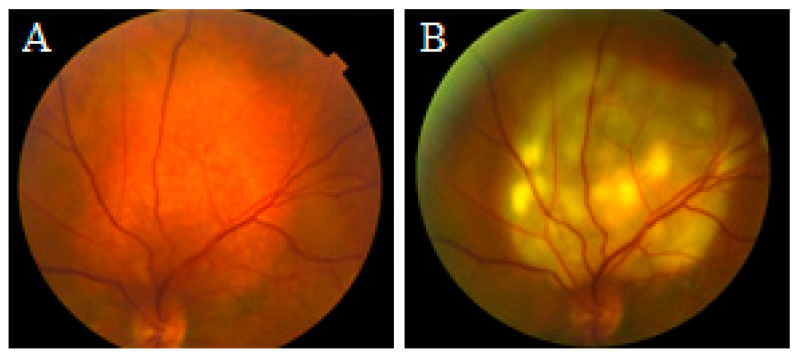
Fundus photograph taken before (**A**) and immediately after LIO-guided transpupillary thermotherapy (TTT) for circumscribed choroidal hemangioma (**B**) showing the response to treatment at 700 mW over 4.5 min.

Seven patients were treated with LIO-guided TTT. Of these, two received treatment at 500–600 mW, two at 700–800 mW, and two at 900 mW power. All patients received a single session of LIO-guided TTT performed under local anesthesia. In the LIO-guided TTT group, the mean applied power was 730 mW (range, 500–900 mW), with a mean exposure duration of 4 min for all patients. The limiting factor for the energy was whitening of the whole surface of the tumor with tolerable or no pain (Figure 1). Indocyanine green (ICG)-enhanced TTT was not utilized in this study.

Six patients were treated by episcleral I-125 plaque brachytherapy. Two patients were treated with 16 mm COMS plaques, delivering an apex dose of 26 Gy and a base dose of 58 Gy, with corresponding foveal, optic disc, and lens doses of 7 Gy, 16 Gy, and 6 Gy, respectively. Two patients received 18 mm COMS plaques, delivering an apex dose of 26 Gy and a base dose of 58 Gy, with corresponding foveal, optic disc, and lens doses of 7 Gy, 16 Gy, and 6 Gy, respectively. The remaining two patients (with tumors touching the optic disc) received 18 mm notched plaques, delivering an apex dose of 24 Gy and a base dose of 56 Gy, with corresponding foveal, optic disc, and lens doses of 35 Gy, 44 Gy, and 3.8 Gy, respectively. Overall, the mean dose to the fovea was 14 Gy (range, 7–35 Gy), to the optic disc was 24 Gy (range, 16–44 Gy), and to the lens was 5 Gy (range, 3.8–6 Gy).

### 3.3. Management Outcome

At the last date of follow-up, visual acuity improved in five out of seven patients (71%) treated with TTT, while none of the six plaque-treated patients demonstrated visual improvement. Vision remained stable in four (67%) plaque-treated and two (29%) TTT-treated patients, while two (33%) patients in the plaque group experienced further visual deterioration. None of the TTT-treated patients experienced visual deterioration (Table 2). The difference in visual outcome between the two groups was statistically significant (*p* = 0.021). When assessing the relationship between tumor location and visual outcomes, we observed that in the TTT group, all three eyes with extramacular tumors, as well as two of four eyes with macular tumors, showed improvement in visual acuity, while the remaining two macular cases maintained stable vision. In contrast, within the plaque group, one eye with an extramacular tumor and three of five eyes with macular tumors achieved stable vision (but no improvement), whereas two of five eyes with macular tumors demonstrated significant deterioration in visual acuity.

OCT imaging was performed in all patients one month following radioactive plaque brachytherapy and LIO-guided TTT to assess subretinal fluid regression; no earlier evaluations were conducted.

**Figure 2:**
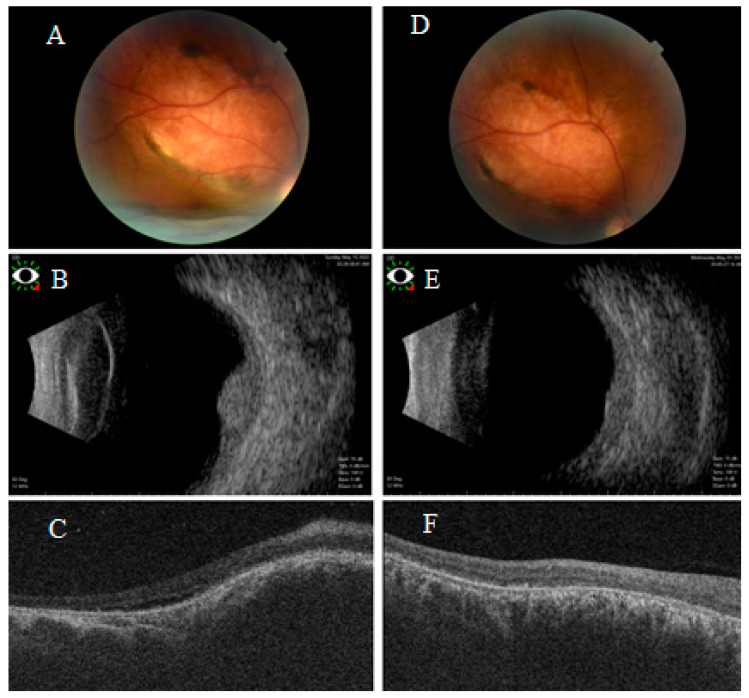
Multimodal imaging of a patient with right circumscribed choroidal hemangioma treated with I-125 plaque brachytherapy. (**A**) Fundus photograph demonstrating right amelanotic choroidal tumor. (**B**) B-scan ultrasonography revealed dome-shaped choroidal lesion with moderate high internal reflectivity and (**C**) OCT confirmed the presence of subretinal fluid. The patient’s best-corrected visual acuity (BCVA) was limited to hand motion before treatment. Post treatment, (**D**) fundus photograph of the treated tumor, with marked regression in thickness confirmed by B scan (**E**) and total resolution of the subretinal fluid confirmed by OCT (**F**). Following therapy, the BCVA did not change (0.02 decimal units).

**Figure 3:**
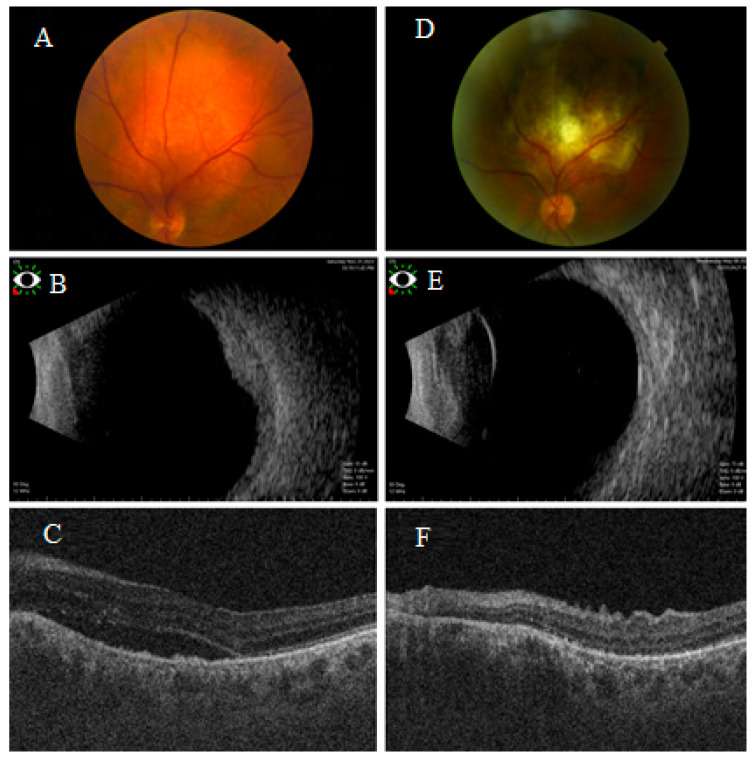
Multimodal imaging of a patient with circumscribed choroidal hemangioma (CCH) treated with laser indirect ophthalmoscope (LIO)-guided transpupillary thermotherapy (TTT). (**A**) Fundus photograph demonstrating left amelanotic choroidal tumor. (**B**) B-scan ultrasonography revealed a dome-shaped choroidal lesion with moderate high internal reflectivity and (**C**) OCT confirmed the presence of subretinal fluid. The patient’s best-corrected visual acuity (BCVA) was 0.5 decimal units prior to treatment. Post-treatment, (**D**) fundus photograph of the treated tumor, with marked regression in thickness confirmed by B scan (**E**) and total resolution of the subretinal fluid confirmed by OCT (**F**). Following therapy, the BCVA improved to 0.8 decimal units.

All patients exhibited a measurable reduction in tumor thickness following treatment. Median tumor thickness reduction was greater in the plaque group (−56%) compared to the TTT group (−36%). In the plaque group median initial thickness was 4.5 mm (range: 4.5–5.0 mm), reduced to 2.5 mm (range: 2.0–3.0 mm). In the TTT group median initial thickness was 3.8 mm (range: 2.9–5.0 mm), reduced to 1.4 mm (range: 0.9–1.8 mm). The difference in tumor thickness reduction between the two groups was statistically not significant (*p* = 1.00) (Figure 2 and Figure 3).

Both treatment groups demonstrated similar efficacy in achieving complete resolution of subretinal fluid, regardless of treatment modality (Figure 2 and Figure 3). None of the patients treated with either plaque brachytherapy or LIO-guided TTT developed recurrence in subretinal fluid over 1 year follow-up (Table 2). No single patient in radioactive plaque group developed cataract or glaucoma, but two patients developed radiation retinopathy. All patients were alive and free of subretinal fluid or tumor recurrence at their last follow-up (median 20 months, mean 24 months, range 12–48 months).

## 4. Discussion

This study demonstrates the efficacy of laser indirect ophthalmoscopy-guided transpupillary thermotherapy (LIO-TTT) in the management of symptomatic circumscribed choroidal hemangioma (CCH) compared with radioactive plaque therapy in the absence of photodynamic therapy (PDT). Both modalities achieved tumor control, reduction in tumor thickness, and complete resolution of subretinal fluid; however, LIO-TTT proved superior in preserving visual function. Subretinal fluid, which is the main cause for vision loss in CCH, improved or resolved in all patients across both treatment groups. This confirms the effectiveness of both modalities in the treatment of macular edema as shown in previous studies [3,6,21]. However, while plaque brachytherapy demonstrated a higher median reduction in tumor thickness (−55.6% vs. −35.9%), it was not associated with any visual improvement. While none of the eyes treated by plaque therapy in this series showed improvement in visual acuity, LIO-TTT resulted in vision improvement in 71% of cases. These findings underscore a critical insight: visual recovery in CCH is not solely determined by the degree of tumor shrinkage, but rather by the treatment’s ability to preserve the surrounding retinal architecture [1,3]. Our results align with previous observations that TTT can offer anatomical and functional benefit, particularly in small-to-medium sized tumors [6,11,18]. However, conventional slit-lamp TTT may lack precision, particularly in eyes with significant subretinal fluid or anterior tumors [21]. LIO-guided TTT, with wide-field visualization and real-time monitoring, improves localization and is both feasible and effective even in challenging cases. Moreover, LIO-TTT is widely available due to its common use in retinoblastoma management.

Peripheral choroidal hemangiomas generally require only observation, as they do not typically affect visual acuity. Treatment is indicated for symptomatic lesions involving the macula or close to the macula and associated with macular edema. When such tumors are treated with radioactive plaque therapy, the radiation dose to the macula will be relatively high, often resulting in radiation retinopathy. In our series, most cases had tumors involving or abutting the fovea, with a mean radiation dose to the fovea of 14 Gy (range, 7–35 Gy) and to the optic disc of 24 Gy (range, 16–44 Gy). These relatively high doses of radiation may cause radiation retinopathy and radiation optic neuropathy, both of which may limit visual recovery in these eyes after treatment. Furthermore, radioactive seeds are not manufactured locally in Jordan and must be imported from overseas, typically causing treatment delays of more than two months. Such delays can lead to further photoreceptor damage due to chronic subretinal fluid or exudative retinal detachment, thereby reducing the potential for visual improvement. Compared to radioactive plaque brachytherapy, LIO-guided TTT has key advantages: it is less invasive (as it does not require surgical implantation of a radioactive plaque), office-based, less expensive, and does not require hospitalization or specialized radiotherapy infrastructure. This makes it especially attractive in resource-limited settings where access to PDT or plaque therapy is restricted [3,7,21].

Historically, PDT has been the preferred modality for symptomatic CCH due to its selectivity and favorable safety profile, offering good tumor regression with minimal retinal damage [3,6,10]. However, PDT requires verteporfin (Visudyne^®^) [6], which remains unavailable in many countries in the Middle East due to limited support from commercial companies. In such settings, plaque brachytherapy has been the default alternative despite its known complications, such as radiation-induced optic neuropathy, maculopathy, and cataract [10]. On the other hand, systemic β-blocker therapy, such as propranolol, has been explored for the treatment of choroidal hemangioma. Krema et al. [27] reported two cases of choroidal hemangioma associated with Sturge–Weber syndrome in which systemic propranolol failed to induce tumor regression, exudative retinal detachment and/or improve visual outcomes, suggesting that β-blockers may have limited therapeutic value in this context. However, its efficacy remains uncertain [28].

According to Shields et al. photodynamic therapy (PDT) is highly effective for circumscribed choroidal hemangioma (CCH). In a series of 79 eyes with unilateral posterior CCH (mean thickness 3.0 mm), 116 PDT sessions were performed with a mean follow-up of 43 months. Good visual acuity (≥20/40) was achieved in 62% of patients, and 93% showed partial or complete resolution of subretinal fluid [18]. Favorable outcomes were associated with smaller tumors, absence of cystoid macular edema, and better baseline vision. A systematic review of 18 studies (221 cases, 2001–2022) further confirmed PDT efficacy. Across studies, baseline best-corrected visual acuity (BCVA) ranged from 20/43 to 20/220, and tumor thickness from 0.6 to 4.1 mm. At final follow-up (1–106 months), mean BCVA improved to 20/20–20/80, and tumor thickness decreased to 0–2.3 mm. Subretinal fluid resolution was reported in 14 studies, and exudative retinal detachment resolution in eight [11]. While PDT is effective, laser indirect ophthalmoscopy-guided transpupillary thermotherapy (LIO-TTT) offers comparable tumor control, comparable visual preservation, and greater accessibility, particularly in regions where verteporfin is unavailable. Even where PDT is accessible, LIO-TTT provides a practical, less invasive, and possibly more cost-efficient alternative, as it avoids intravenous photosensitizers and their systemic risks [3,6,19,21]. Gündüz et al. (2021) [21] reported cases of circumscribed choroidal hemangioma, where final visual acuity (VA) ≤ 20/200 occurred in 48.0% of TTT-treated eyes, 13.6% of ICG-TTT-treated eyes, and 30.8% of PDT-treated eyes (*p* = 0.041), while for <2 Snellen lines VA improvement was observed in 64.0%, 27.3%, and 38.5% of eyes, respectively (*p* = 0.036). Decrease in tumor thickness by <20% was linked to smaller baseline tumor size (*p* = 0.045) and retinoschisis (*p* = 0.014). This study showed that longer symptom duration, previous failed treatment, lower initial VA, and presence of retinoschisis were risk factors for worse visual outcomes. And in another cohort with a mean age of 36 years, circumscribed choroidal hemangioma (CCH) was sub-foveal in 37.5% of eyes, with exudative retinal detachment present in all cases. Tumor measurements showed a median thickness of 4.05 mm. Following TTT, visual acuity improved in 44%, remained stable in 37%, and worsened in 19% of eyes. Resolution of exudative retinal detachment was achieved in 94% of eyes, with a median follow-up duration of 9.5 months [22].

We believe our study is unique, being the first to use LIO-guided TTT, rather than the conventional slit-lamp-based TTT approach, for the management of choroidal hemangioma—A technique readily available in most ocular oncology centers, as it is routinely used in the treatment of retinoblastoma. However, our study has several limitations, including its retrospective design, small sample size, and single-center nature, which may introduce selection bias. A potential concern in this study could be selection bias; however, the allocation of patients to treatment groups was determined solely by the historical availability of therapies at our institution. Initially, plaque brachytherapy was the standard treatment for choroidal hemangioma because it was the only available treatment modality and PDT was not available, and later, LIO guided transpupillary thermotherapy (TTT) was approved in our institution for this indication and therefore was adopted to assess its efficacy and visual outcomes. Therefore, patients received treatment according to the prevailing therapeutic approach at the time, rather than any clinical selection criteria, minimizing the risk of bias related to patient characteristics. Despite these limitations, the statistically significant difference in visual outcomes strongly supports the utility of LIO-guided TTT. Future prospective, multi-center studies with larger sample sizes and longer follow-up are needed to validate these findings and determine optimal patient selection criteria.

## 5. Conclusions

Laser indirect ophthalmoscopy-guided transpupillary thermotherapy (LIO-guided TTT) represents an effective and accessible treatment modality for circumscribed choroidal hemangioma. It demonstrated significantly superior visual outcomes compared to plaque brachytherapy, despite both treatments achieving comparable anatomical tumor regression and subretinal fluid resolution. These findings underscore the critical importance of choosing treatment approaches that not only control tumor growth (as it is a benign tumor) but also prioritize visual function. Given its noninvasive nature, low cost, and ease of implementation, LIO-guided TTT has the potential to become a first-line treatment, particularly in regions where photodynamic therapy is unavailable. Further prospective studies with larger cohorts are warranted to validate its long-term efficacy and define its role in global ocular oncology practice.

## Figures and Tables

**Table 1:** Demographics of 13 patients diagnosed with choroidal hemangioma and treated by TTT or I-125 plaque brachytherapy.

		Whole Group (N ^#^, %)	TTT * Group (N, %)	Plaque Group (N, %)	*p* Value
Number		13 (100%)	7 (54%)	6 (46%)	
Age ^@^ (Median, Mean, Range)		42, 43, 4–62	40, 36, 4–58	47, 49, 38–62	
Gender	Male	10 (77%)	6 (60%)	4 (67%)	0.559
	Female	3 (33%)	1 (33%)	2 (33%)	
Side	Right	5 (38%)	3 (43%)	2 (33%)	1.00
	Left	8 (62%)	4 (57%)	4 (67%)	
Visual Acuity at diagnosis	<0.1	6 (46%)	2 (29%)	4 (67%)	0.286
	0.1–0.5	7 (54%)	5 (71%)	2 (33%)	
	>0.5	0 (0%)	0 (0%)	0 (0%)	
Tumor Thickness	<3 mm	1 (8%)	1 (14%)	0 (0%)	1.00
	3–5 mm	12 (92%)	6 (86%)	6 (100%)	
Site	Macular	9 (69%)	4 (57%)	5 (83%)	0.559
	Extra-macular	4 (31%)	3 (43%)	1 (17%)	
Subretinal fluid	Yes	13 (100%)	7 (100%)	6 (100%)	1.00
	No	0 (0%)	0 (0%)	0 (0%)	

* TTT: Transpupillary thermal therapy. ^#^ Number. ^@^ Age in years.

**Table 2:** Outcome of management for 13 eyes with choroidal hemangioma treated by TTT * or I-125 plaque brachytherapy.

		TTT * Group (N ^#^, %)	Plaque Group (N, %)	*p* Value
Number		7 (54%)	6 (46%)	
Vision after therapy	<0.1	2 (29%)	4 (67%)	0.027
	0.1–0.5	3 (42%)	2 (33%)	
	>0.5	2 (29%)	0 (0%)	
Change in Vision	Improved	5 (71%)	0 (0%)	0.016
	stable	2 (29%)	4 (67%)	
	Deteriorated	0 (0%)	2 (33%)	
Thickness after therapy	<3 mm	6 (86%)	6 (100%)	1.00
	3–5 mm	1 (14%)	0 (0%)	
Change in Thickness	Improved	7 (100%)	6 (100%)	1.00
	No Improvement	0 (0%)	0 (0%)	
Subretinal fluid	Improved	7 (100%)	6 (100%)	1.00
	No Improvement	0 (0%)	0 (0%)	
Recurrent Fluid	Yes	0 (0%)	0 (0%)	1.00
	No	7 (100%)	6 (100%)	
Survival	Yes	7 (100%)	6 (100%)	1.00
	No	0 (0%)	0 (0%)	

* TTT: Transpupillary thermal therapy. ^#^ Number.

## Data Availability

The research data is available upon reasonable request.
